# 126. Outpatient Antimicrobial Stewardship Utilizing a Decentralized Model

**DOI:** 10.1093/ofid/ofab466.328

**Published:** 2021-12-04

**Authors:** Harry Powers, Stephen McMullan, Therese Anderson, Deborah Boeff, William Bonner, Kevin L Epps, Dana Harris, Jacqueline LaCouture, Michelle A Leak, Angelica Leybeg, Izabela Riffe, Lynda Schnusenberg, Julio C Mendez

**Affiliations:** 1 Mayo Clinic Florida, Jacksonville, Florida; 2 mayo clinic, Ponte Vedra, Florida; 3 Mayo Clinic, Atlantc Beach, Florida

## Abstract

**Background:**

The majority of human antimicrobial utilization occurs in the outpatient setting. Despite being mainly viral in etiology, upper respiratory tract infections (URIs) were the most common indication for outpatient antimicrobial prescriptions at our institution.

**Methods:**

Through our electronic health record (EHR), we were able to determine our rate of antibiotic prescriptions for inappropriate URI diagnosis at our primary care practice sites. We selected staff volunteers from each our primary care practice sites to serve as stewardship champions. They were given training in stewardship best practices, and an URI stewardship toolkit which included viral URI prescription pad, EHR order panel, and patient education signage. They were tasked with providing education and feedback to their practice sites. We meet with them on a monthly basis to disseminate prescribing data and education. They also provided feedback from practice sites to the stewardship committee.

**Results:**

Our decentralized model was put in place in November 2020. In the 6 months prior to the intervention, the average prescribing rate was 29.1%. In the 6 months after the intervention, the average prescribing rate decreased by 15% to 24.8%. During the intervention phase, there was an increase in number of non-COVID URIs diagnosed at our primary care sites.

Temporal Trend in Inappropriate Antibiotics Prescribing Rates for Viral URIs Pre- and Post- Intervention

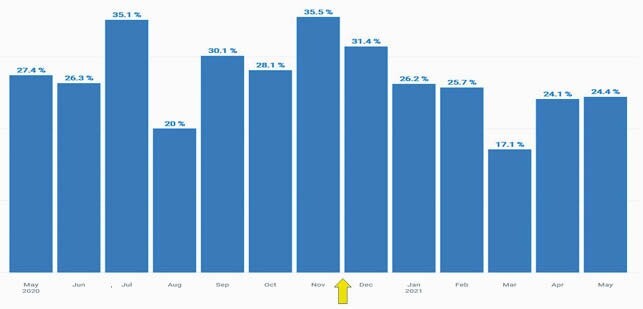

Inappropriate antibiotic prescribing rate for viral upper respiratory tract infections from May 2020 until May 2021. Intervention started in December 2021 (arrow). Pre-intervention average was 29.1%. Post-intervention age was 24.8% which is a 15% decline in prescribing rate.

Viral Upper Respiratory Infections Visits

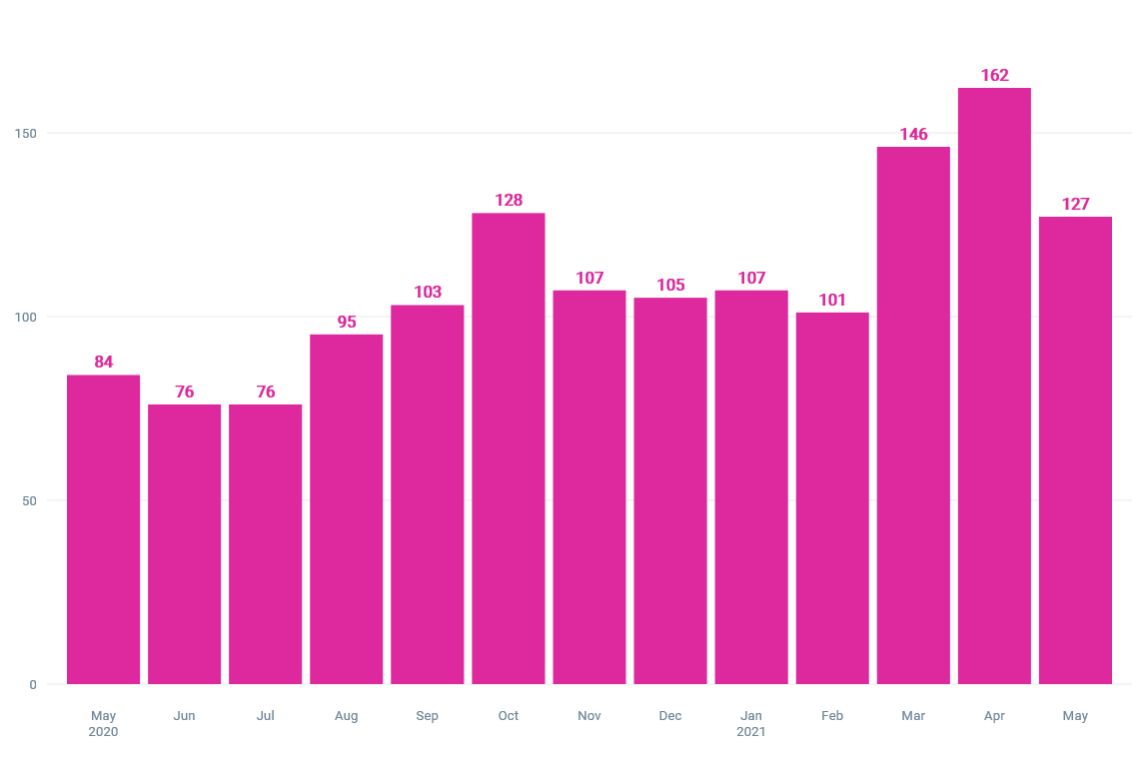

The total number of visits for presumed viral upper respiratory infections to primary care sites from May 2020 until May 2021. The majority of COVID-19 precautions in the area expired at the end of March 2021.

**Conclusion:**

We have been able to lower our inappropriate prescriptions for URIs utilizing a decentralized model of stewardship champions. This result was especially notable as the intervention phase corresponded with the end of COVID-19 precautions and an increase in non-COVID URIs diagnosed. The advantage of this approach includes an advocate embedded at each practice site who is familiar with the opportunities and challenges of the site, and a two-way flow of information from practice sites to the stewardship committee. This model provided additional benefit during the COVID-19 pandemic as the ability of centralized staff to travel to off campus clinic sites was curtailed.

**Disclosures:**

**All Authors**: No reported disclosures

